# Ultrafast synthesis of Au(I)-dodecanethiolate nanotubes for advanced Hg^2+^ sensor electrodes

**DOI:** 10.1186/1556-276X-9-601

**Published:** 2014-11-05

**Authors:** Zhiqiang Zhang, Congcong Ma, Lian He, Shijin Zhu, Xiaodong Hao, Wanyi Xie, Wei Zhang, Yuxin Zhang

**Affiliations:** 1 College of Material Science and Engineering, Chongqing University, Chongqing 400044, People’s Republic of China; 2 Key Laboratory of Multi-scale Manufacturing Technology, Chongqing Institute of Green and Intelligent Technology, Chinese Academy of Sciences, Chongqing 400714, People’s Republic of China; 3 National Key Laboratory of Fundamental Science of Micro/Nano-Devices and System Technology, Chongqing University, Chongqing 400044, People’s Republic of China

**Keywords:** Functional, Nanocomposite, Multilayer structure, Self-assembly, Sensor

## Abstract

In this work, an ultrafast and facile method is developed to synthesize Au(I)-dodecanethiolate nanotubes (Au(I)NTs) with the assistance of glycyl-glycyl-glycine (G-G-G). Transmission electron microscopy (TEM) images reveal that the as-prepared Au(I)NTs can be obtained in a 2-h reaction instead of a previous 24-h reaction and are uniform with a hollow structure and smooth surface by virtue of the G-G-G peptide tubular template. According to structural analysis, a possible preparative mechanism is proposed that the G-G-G peptide could help to curl into tube-like morphology in alkaline situation spontaneously to accelerate the formation of Au(I)NTs. Meanwhile, PVDF-stabilized Au(I)NT-modified glassy carbon electrodes present their promising potential for Hg^2+^ detection.

## Background

Nanotubes have become an area of increasing interest because of their wide application in nanotechnology [1-5]. Gold nanotubes (AuNTs) are paid attention due to their unique optical and electrical properties in surface-enhanced Raman spectra (SERS)-active substrate [6] and as a refractive index reporter [7]. Up to now, one of the popular methods is hard template approach, using Ag nanowires [8], Ni nanorods [9], polymer nanowires [10], and Co nanoparticles (CoNPs) [11] as sacrificial templates. These templates are usually synthesized in the anodizing aluminum oxide (AAO) template first, since the pore diameter and pore arrangement of AAO can be tuned and controlled. However, these templating materials have to be removed via accurate control so that the conditions would be complex, and the environment would be polluted by using some strong acid, alkali, or oxidant. Recently, Zhang et al. reported a unique and green strategy to fabricate Au(0) nanotubes using Au(I)NTs as seed precursor [12]. They found that when HAuCl_4_ met 1-dodecanethiol in the strong alkali and stirring condition, the hybrids had a tendency to curl into tubes, followed by reduction into Au(0)NTs under electron beam irradiation. However, the self-assembly of Au(I)-alkanethiolate nanotubes would cost at least 20 h, limiting its wide application in biosensors and chemical sensors. With regard to accelerating the formation process of this special nanotube, some efforts have been put through.

As we know, cyclic peptide favored the self-assembly synthesis of organic nanotubes [13,14]. This kind of organic nanotubes can serve as the building block to assemble to nanometer-scale devices [15-19] due to their unique advantages such as the mild synthesizing condition, low cost, and environment friendly. Herein, we combine the self-assembly of Au(I)-organic with the peptide nanotube to successfully synthesize Au(I) nanotube in a very short time. According to our recent work [20], this kind of novel Au(I) nanotubes also present potential selectivity for Hg^2+^ detection, as well as other Au nanostructures [21-24].

## Methods

All the chemicals including aqueous hydrogen tetrachloroaurate trihydrate (HAuCl_4_ · 3H_2_O, 99.99%, Alfa Aesar, Ward Hill, MA, USA), 1-dodecanethiol (DDT, C_12_H_25_SH, 98%, Alfa Aesar), glycyl-glycyl-glycine (G-G-G, C_6_H_11_N_3_O_4_, 99%, Alfa Aesar), sodium hydroxide (NaOH, 98%, Chuandong Chemical, Chongqing, China), and Hg(NO_3_)_2_ (98%, Chuandong Chemical) were used without further purification.

The Au(I)-dodecanethiolate nanotubes were synthesized by a modified method [12]. In a typical synthesis, the reaction mixture containing 1 mL of DDT ethanol solution (0.1 M) was added to 1 mL of aqueous HAuCl_4_ (0.01 M) under static condition. Then, 1 mL of NaOH solution (1 M) was added dropwise to the above solution under vigorous stirring. Subsequently, 1 mL of G-G-G aqueous solution (0.1 M) was injected into the solution. The resulting mixture was maintained at room temperature under vigorous stirring condition for 2 h. The products were collected by centrifugation, followed by washing with ethanol for three times. Finally, the gold samples obtained were re-dispersed in ethanol.

The structural and morphological investigations of the samples were carried out by high-resolution transmission electron microscopy (HRTEM; ZEISS LIBRA 200, 200 kV, Carl Zeiss AG, Oberkochen, Germany). The crystallographic information and chemical composition of the as-prepared products were established by powder X-ray diffraction (XRD; D/max2500, Cu Kα). The presence of 1-dodecanthiolate in Au(I)-SC_12_H_25_ nanotubes was confirmed with Fourier transform infrared spectroscopy using the KBr pellet method (FTIR; Nicolet 5DXC FT-IR). The percentage of the organic phase in the hybrid nanotubes was analyzed by the thermogravimetric analysis (TGA; NETZSCH STA 449 F3, NETZSCH, Shanghai, China) under Ar atmosphere.

As for the Hg^2+^ sensor application, a bare glass carbon electrode (GCE) was first polished to a mirror-like surface with 0.3 and 0.5 μm alumina powder followed by rinsing thoroughly with deionized water, then sonicated in 1:1 nitric acid (*v*/*v* for HNO_3_/H_2_O) and deionized water, and then dried at room temperature. The cleaned electrode was modified with PVDF-stabilized Au(I)NTs by a simple casting method. Typically, 0.5 wt% nafion solution (5 μL) and prepared Au(I)NT solution (10 μL) were mixed first, then cast on the electrode surface, and dried at room temperature to obtain a PVDF-Au(I)NT-modified electrode. Hg^2+^ work solution was prepared in 0.1 M HCl solution and then accumulated at -0.60 V for 500 s while stirring the solution. The solution was then left for 30 s, and the square wave anodic stripping voltammetry (SWASV) measurements were performed in the potential range from -1.0 to +0.60 V with a frequency of 30 Hz, amplitude of the square wave of 25 mV, and a potential step of 4 mV. The peak heights were measured at -0.20 V.

## Results and discussion

**Figure 1:**
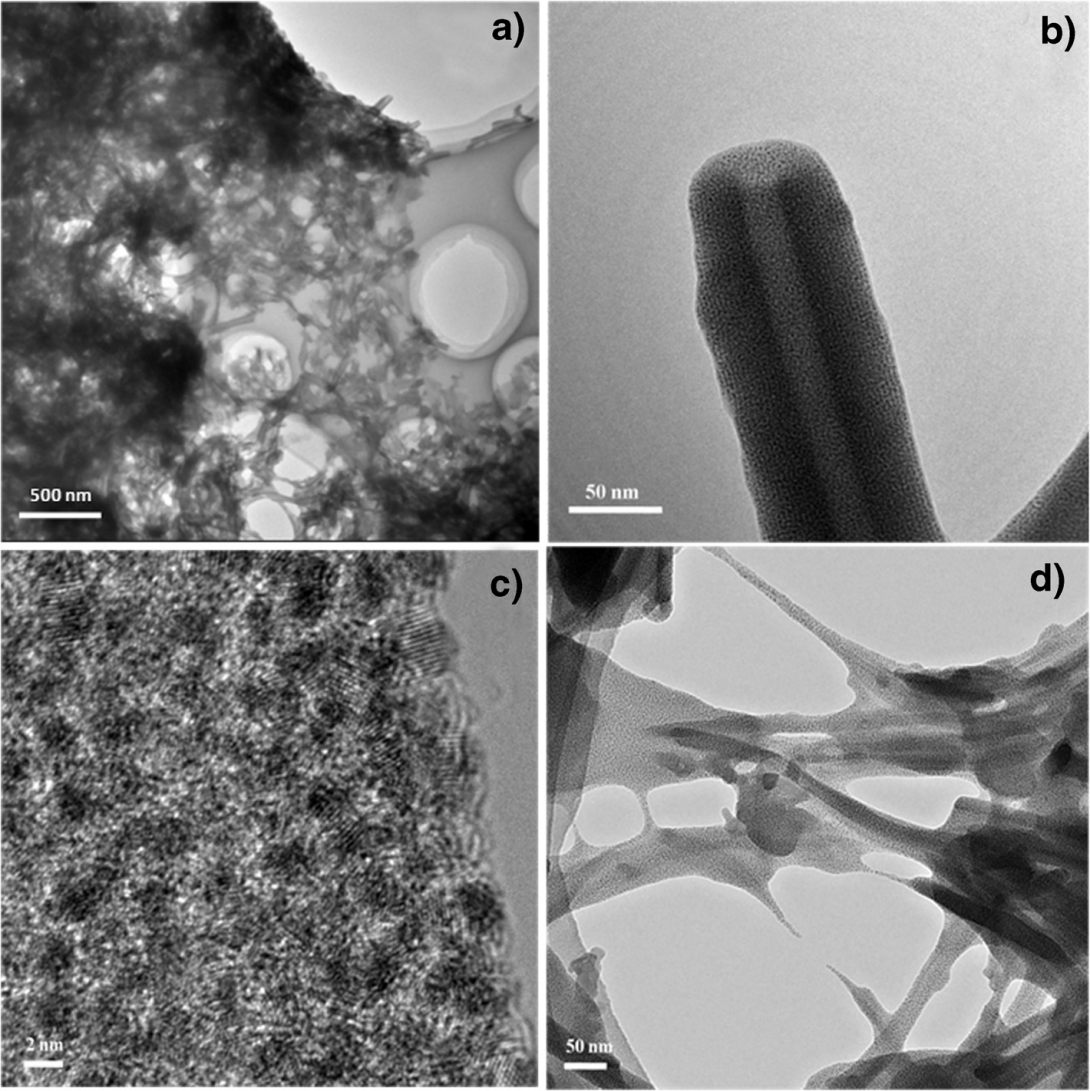
**TEM images. (a, b)** TEM images of the as-prepared Au(I)-dodecanethiolate nanotubes with gly-gly-gly in 2 h. **(c)** HRTEM image of the as-prepared Au(I)-dodecanethiolate nanotubes. **(d)** TEM image of the products without gly-gly-gly in 2 h.

Figure 1 presents the representative TEM images of the as-prepared Au(I)-dodecanethiolate nanotubes with or without gly-gly-gly. Detailedly, Figure 1a displays a macroscopical view of uniform nanotube formation without any nanosheet, indicative of an enhancive yield [12]. Figure 1b reveals that the nanotubes have smooth surface and narrow diameter distribution with 100 nm in diameter. Since Au(I) compounds rest with beam sensitive matter, Au(I)NTs could be reduced into Au(0) nanotubes, with obvious gold nanoparticle morphology and uniform distribution (approximately 2 nm; Au^+^ can be deoxidated to Au^0^ by the electron beam) [12]. The thickness of the walls is in the range of ca. 35 nm, and the ends of the nanotubes are always open). Figure 1c shows the HRTEM image of the as-prepared Au(I)-dodecanethiolate nanotubes and the well-resolved lattice fringe which is consistent with the XRD patterns. In comparison, there are only mesoporous Au(I) nanosheet without the assistance of G-G-G in initial 2 h (see Figure 1d). This porous network has sparse curling nanosheet, presenting the important role of surfactant ligands such as gly-gly-gly.

**Figure 2:**
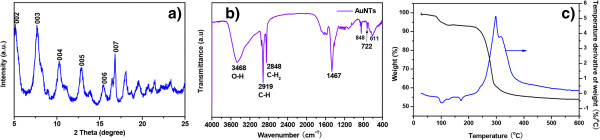
**XRD patterns, FTIR spectrum, and TGA spectrum. (a)** XRD patterns, **(b)** FTIR spectrum, and **(c)** TGA spectrum of the as-prepared Au(I)-dodecanethiolate nanotubes with gly-gly-gly in 2 h.

To further reveal the chemical constitution and structure of the hybrid nanotubes, XRD patterns were obtained in Figure 2a, presenting that there is no Au(0) existence in the nanotube as there is no XRD signal of the Au(0). The periodic diffraction indicates the bilayer structure of the nanotubes, and the nearest distance between two adjacent walls is in the range of 3.384 ± 0.010 nm, in accord with our previous work [12]. This finding also verifies that gly-gly-gly does not tune the distance of Au(I)-thiol bilayer structures.

The FTIR spectrum of the nanotubes (see Figure 2b) shows the general fingerprint features of linear dodecanethiolate, confirmed by the absence of the absorption band of ν(S-H) at 2,526 cm^-1^. The TGA analysis of the nanotubes (see Figure 2c) indicates that the organic phase in the nanotubes has a population of about 40.66%.

**Figure 3:**
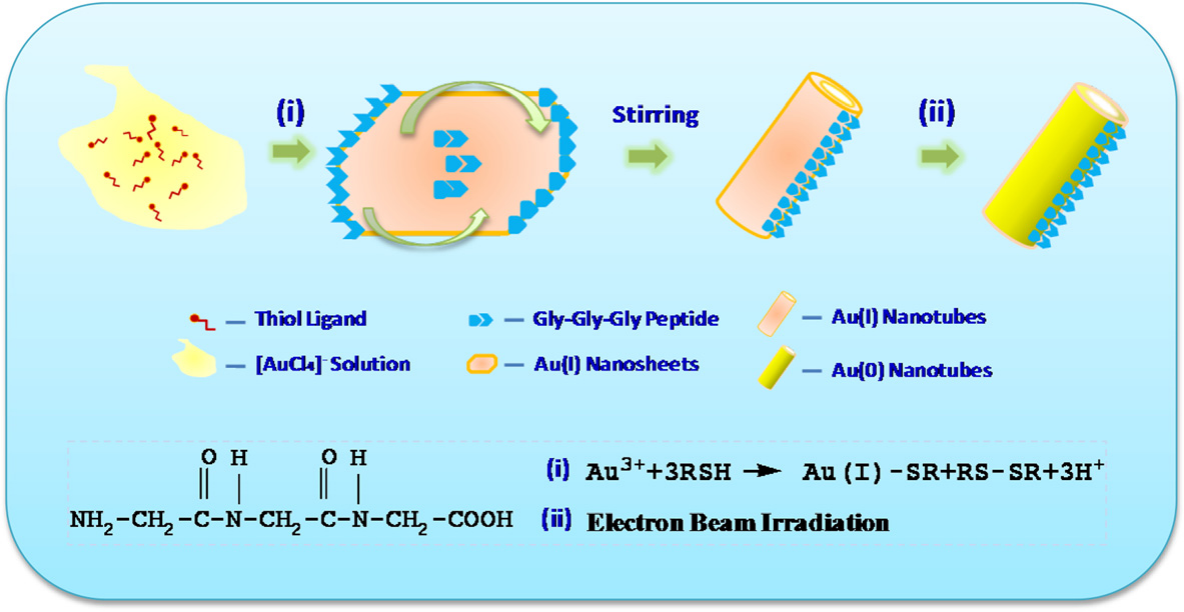
**Schematic illustrations.** Schematic illustrations of the as-prepared Au(I)-dodecanethiolate nanotubes with gly-gly-gly in 2 h.

On the basis of the above observation, the possible ultrafast formation mechanism of the Au(I)-alkanethiolate nanotubes is shown in Figure 3. We conclude that 1) the hybrid of HAuCl_4_ and DDT can form nanotubes through self-assembly method; the reaction equation is Au^3+^ +3RH → Au(I)-SR + RS-SR +3H^+^[19], but this process requires a longer time (stirring for at least 20 h). 2) The G-G-G peptides behave like a surfactant, which can easily build into tubular structures via hydrogen bonding (‘suspended’ ligands). The ligands connect each other and serve as the soft tubular-like template to synthesize the gold-organic nanotubes. The G-G-G peptides can accelerate the formation of Au(I) nanotubes, instead of doping or intercalation in the bilayer structure.

**Figure 4:**
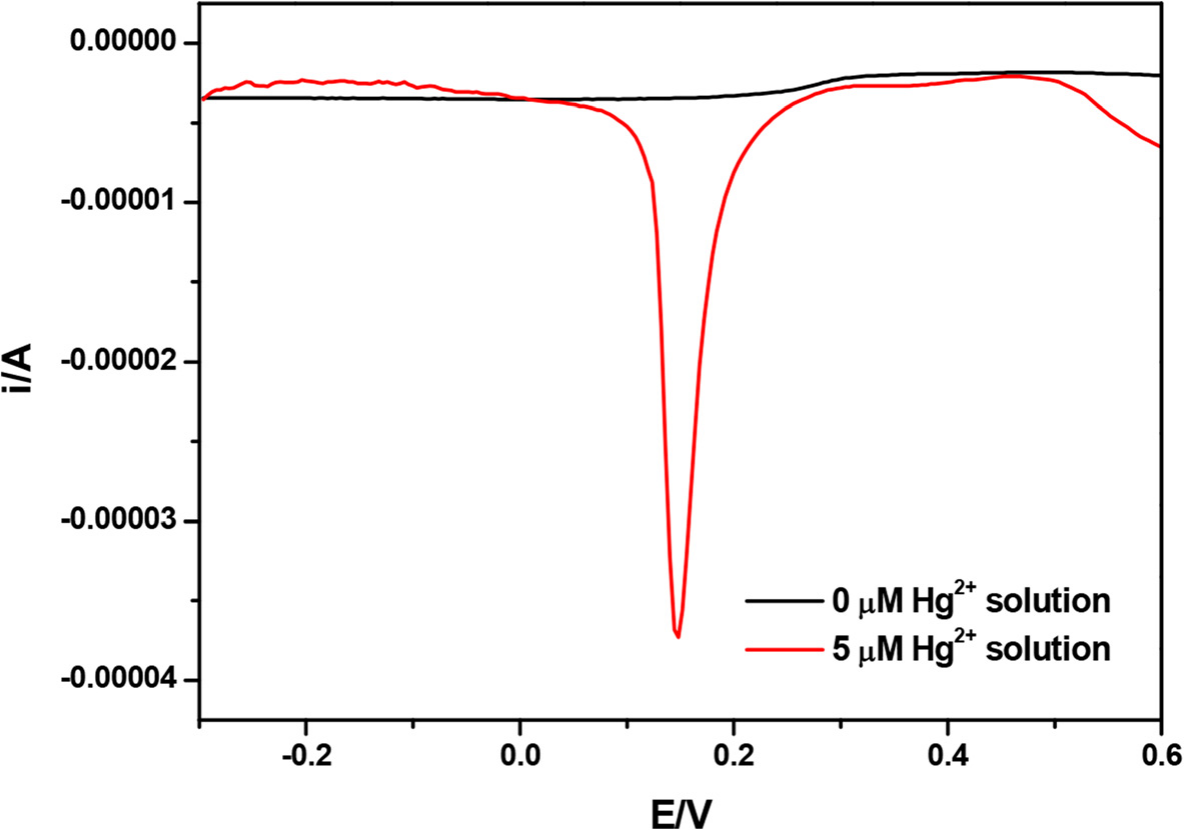
**Square wave anodic stripping voltammetry.** SWASV response for 0 and 5 μM Hg^2+^ in 0.1 M HCl. SWASV conditions: quiet time, 10 s; frequency, 30 Hz; potential increment, 4 mV; amplitude of the square wave, 25 mV.

In order to elucidate the practical application of Hg^2+^ sensor, SWASV response is examined, as shown in Figure 4, indicating that the adsorbed Hg^2+^ was reduced to Hg^0^ at a certain potential. The SWASV analytical characteristics of bare GCE and PVDF-Au(I)NT-modified GCE present different responses in 5 μM Hg^2+^ in 0.1 M HCl solution. The sharper and higher peak current for Hg^2+^ is obtained at the PVDF-Au(I)NT-modified GCE, compared with the bare GCE, and this phenomena is consistent with the previous work [20].

## Conclusions

In this work, we have developed a very quick self-assembly method to synthesize Au(I) nanotubes from 24 to 2 h via introducing G-G-G. The as-prepared Au(I) nanotubes have a uniform morphology of bilayer structure of Au-SC_12_H_25_ and well-distributed diameters with the help of G-G-G tubular template. Intriguingly, G-G-G peptide-assisted Au(I) nanotubes also demonstrate their selective sensor response of Hg^2+^ detection.

## Abbreviations

AAO: anodic aluminum oxide; Au(I)NTs: Au(I)-dodecanethiolate nanotubes; AuNTs: gold nanotubes; CoNPs: Co nanoparticles; DDT: 1-dodecanethiol; G-G-G: glycyl-glycyl-glycine; HAuCl_4_ · 3H_2_O: aqueous hydrogen tetrachloroaurate trihydrate; NaOH: sodium hydroxide; SERS: surface-enhanced Raman spectra; SI: supporting information; TEM: transmission electron microscopy.

## Competing interests

The authors declare that they have no competing interests.

## Authors’ contributions

ZZ and YZ synthesized and characterized the Au(I)-dodecanethiolate nanotubes and wrote the manuscript. CM and LH prepared the advanced Hg^2+^ sensor electrodes. SZ and XH conceived and designed the experiments. WX and WZ coordinated the study. All authors read and approved the final manuscript.
